# Association between Physical Activity and 32 Chronic Conditions among Spanish Adults

**DOI:** 10.3390/ijerph192013596

**Published:** 2022-10-20

**Authors:** Guillermo F. López Sánchez, Jaime Mendiola Olivares, Alberto M. Torres Cantero

**Affiliations:** Division of Preventive Medicine and Public Health, Department of Public Health Sciences, School of Medicine, University of Murcia, 30120 Murcia, Spain

**Keywords:** physical activity, chronic conditions, chronic diseases, noncommunicable diseases, Spain

## Abstract

The objective was to analyse the association between physical activity and the risk of suffering from 32 chronic conditions using a large representative sample of Spanish adults. We utilised the dataset of the last edition of the Spanish National Health Survey, which was conducted in the year 2017. This dataset included a total of 23,089 adults between the ages of 15 and 103 years. The average age was 53.4 years (standard deviation 18.9 years). Regarding sex distribution, 54.1% of the participants were females. The instrument used to measure physical activity was the short form of the international physical activity questionnaire. The question used to evaluate if the participants suffered from chronic conditions was “Have you ever been diagnosed with chronic condition?”. This question was asked for 32 different chronic conditions. The association between low levels of physical activity (exposure) and chronic conditions (outcome) was assessed with multivariable logistic regression analyses. The highest prevalence of chronic conditions was found in the group doing less than 600 MET (metabolic equivalent of task)-min/week of physical activity (in 28 of the 32 conditions analyzed). The lowest prevalence was in the group doing at least 1200 MET-min/week (in 30 of the 32). Adjusted multivariable logistic regression analyses showed that less than 600 MET-min/week of physical activity was significantly associated with a higher risk of 19 chronic conditions. All these significant associations, except for hypertension, were also maintained in those doing less than 1200 MET-min/week. In conclusion, higher physical activity is a protective factor against the risk of suffering from chronic conditions, with the lowest prevalence of chronic conditions in people doing more than 1200 MET-min/week. International physical activity guidelines should recommend at least 1200 MET-min/week to prevent the risk of chronic conditions.

## 1. Introduction

Chronic conditions, also known as noncommunicable diseases (NCDs), tend to be long in duration and result from a combination of genetic, physiological, environmental, and behavioural factors [1]. Chronic conditions kill 41 million people each year, equivalent to 71% of all deaths globally, and each year more than 15 million people die from a chronic condition between the ages of 30 and 69 years [1]. In Spain, it has been estimated that chronic conditions are the cause of 92% of the total number of deaths in the country, with the majority of them cardiovascular diseases (31%) and cancers (28%), and with a probability of 11% of dying between the ages of 30 and 70 due to the four main chronic diseases (cancer, diabetes, cardiovascular disease, and chronic respiratory disease) [2]. 

Important risk factors contributing to the appearance of chronic conditions are unhealthy lifestyles, such as physical inactivity, unhealthy diets, exposure to tobacco smoke, or the harmful use of alcohol [3]. Therefore, an important way to control chronic conditions is to focus on reducing these risk factors. Specifically, physical activity has significant health benefits for hearts, bodies, and minds and contributes to preventing and managing chronic conditions, such as cardiovascular diseases, cancer, diabetes, depression, and anxiety [4].

According to World Health Organization (WHO) recommendations, adults living with chronic conditions (hypertension, type 2 diabetes, HIV (human immunodeficiency virus), and cancer survivors) should do the same amount of weekly physical activity as healthy adults: at least 150–300 min of moderate-intensity aerobic physical activity or at least 75–150 min of vigorous-intensity aerobic physical activity or an equivalent combination of moderate- and vigorous-intensity activity throughout the week [4]. In terms of MET (metabolic equivalent of task), this would be equivalent to 600–1200 MET-min/week of physical activity [4,5,6]. However, more than a quarter of the world’s adult population (1.4 billion adults) are insufficiently active, with around one in three women and one in four men not achieving these recommended physical activity levels [4]. In Spain, according to a study analyzing physical activity among 17,777 Spanish adults, the overall prevalence of Spanish adults doing less than 600 MET-min/week of physical activity is 30.2% [7].

Previous studies analysing the association between physical activity and chronic conditions have focused on the main types of chronic conditions: cardiovascular diseases, cancers, chronic respiratory diseases, diabetes, and mental health conditions [4,8,9]. However, to our knowledge, there are no studies analysing the association between physical activity and a complete list of chronic conditions in a large representative sample of adults, which would allow comparing the impact of physical activity levels on different chronic conditions. Therefore, the objective of this study was to analyse the association between physical activity and the risk of suffering from 32 chronic conditions in a large representative sample of Spanish adults. Considering that previous research has found important benefits of physical activity for health in different populations [4,10,11] and that each chronic condition is different, it was hypothesised that low levels of physical activity would be associated with a higher risk of suffering from all chronic conditions, but with a different impact of physical activity on each chronic condition. The results of this study will allow for the design of specific public health strategies and interventions for different chronic conditions.

## 2. Methods

### 2.1. The Survey 

The present study utilised data from the last edition of the Spanish National Health Survey (2017), with data collection taking place between October 2016 and October 2017. Therefore, the design of this study was cross-sectional. A detailed description of the Spanish National Health Survey is provided in previous literature, and here we provide a brief overview [12,13]. A three-stage stratified sampling method was employed for data collection. In the first stage, census sections were taken into consideration; in the second stage, the family dwellings, and in the third stage, an adult (older than 15 years) was chosen within each dwelling. The probability was proportional to the size to select the sections in each stratum. Regarding the dwellings, they were chosen with equal probability in each section using systematic sampling, prior arrangement considering the dwelling size. Therefore, due to this process, samples were self-weighted in each stratum. In order to select the person who had to answer the adult questionnaire, the random Kish method was applied, which assigns the same probability to all adults older than 15 years living in the household. A total of 23,089 adults (age range: 15–103 years; average age: 53.4 ± 18.9 years; 54.1% females) participated in this survey, allowing for a representative sample of the adult Spanish population. The method that the Spanish Health Survey used to collect the data was CAPI (computer-assisted personal interviewing). The personal interviews took place in the houses of the respondents. All the interviewers had previously received specific training about the CAPI method. All respondents signed an informed consent form before proceeding with the personal interviews of the survey. All the principles of the Declaration of Helsinki (of the World Medical Association) were followed in the survey and in this study. According to European legislation, public datasets do not need ethical approval when they are used for statistical analyses and research.

### 2.2. Physical Activity (Exposure)

The instrument used to measure the level of physical activity was the short form of the international physical activity questionnaire, also known as IPAQ. MET-min/week is the specific unit utilised by the IPAQ to quantify physical activity. MET stands for the metabolic equivalent of task. This questionnaire uses a formula to calculate the total MET-min/week of physical activity, which consists of the sum of walking and moderate and vigorous MET-min/week scores [14]. Based on the lower and upper limits of the WHO physical activity guidelines [4,5,6], physical activity was ategorized as low (<600 MET-min/week), medium (600 to <1200 MET-min/week), and high (≥1200 MET-min/week). Adults aged ≥70 years did not complete the IPAQ short form, as this questionnaire was developed for population surveillance of physical activity among adults aged 15−69 years, and its use with older and younger age groups is not recommended [14]. IPAQ has been validated in adult populations from different countries showing acceptable validity (ρ = 0.30, 95% CI: 0.23–0.36) and reliability (Spearman’s ρ = 0.81, 95% CI: 0.79–0.82) [5]. Specifically, the IPAQ short form has been validated among Spanish university students showing adequate validity [15]. 

### 2.3. Chronic Conditions (Outcome)

A total of 32 different chronic conditions were analysed in the present study, based on the data available in the Spanish National Health Survey 2017. Participants who answered affirmatively to the question “Have you ever been diagnosed with “chronic condition?” were determined to suffer from that specific condition. The chronic conditions studied were: 1. Hypertension; 2. Myocardial infarction; 3. Angina, Coronary heart disease; 4. Other heart diseases; 5. Varicose veins (legs); 6. Arthrosis; 7. Chronic back pain (cervical); 8. Chronic back pain (lumbar); 9. Chronic allergy (allergic asthma excluded); 10. Asthma (allergic asthma included); 11. Chronic bronchitis, emphysema, COPD; 12. Diabetes; 13. Stomach/duodenal ulcer; 14. Urinary incontinence; 15. High cholesterol; 16. Cataracts; 17. Chronic skin problems; 18. Chronic constipation; 19. Cirrhosis, liver dysfunction; 20. Depression; 21. Chronic anxiety; 22. Other mental problems; 23. Stroke; 24. Migraine or frequent headache; 25. Haemorrhoids; 26. Malignant tumours; 27. Osteoporosis; 28. Thyroid problems; 29. Kidney problems; 30. Prostate problems (males); 31. Menopausal problems (females); 32. Permanent injuries caused by accident. 

### 2.4. Covariates

The selection of covariates was based on past literature [16,17,18,19,20,21,22,23,24]. Sociodemographic variables included gender, age, education level, and living as a couple. Education was based on the highest educational level achieved and was categorized as ≤primary, secondary, and ≥tertiary. Living as a couple was categorized as yes/no. Smoking status was self-reported and categorized as never, current smoker, and past smoker. Alcohol consumption in the last 12 months was self-reported and categorized as yes (any) and no (none). Height and weight were self-reported, and body mass index (BMI) was calculated as weight in kilograms divided by height in meters squared. 

### 2.5. Statistical Analysis

SPSS 25.0 (International Business Machines Corporation, New York, NY, USA) was the statistical software used to carry out the statistical analyses. The prevalence (frequency and percentage) of each of the 32 chronic conditions analysed in the sample of adults living in Spain was calculated by physical activity levels (<600 MET-min/week; 600 to <1200 MET-min/week; ≥1200 MET-min/week). Multivariable logistic regression analyses were then conducted to measure the association between low levels of physical activity (exposure) and chronic conditions (outcome) in Spanish adults. The cutoffs used to establish the physical activity levels in this association were based on the recommendations and physical activity guidelines of the WHO [4,5,6]: <600 MET-min/week and <1200 MET-min/week. Regression models were adjusted for several important covariates according to previous literature, including sociodemographic characteristics (gender, age, education level, and the fact of living as a couple) and other health parameters (BMI, smoking, and alcohol consumption) [16,17,18,19,20,21,22,23,24]. In the analyses, the variables were treated as categorical variables, except the variables age and BMI, which were treated as continuous variables. The results obtained after conducting the logistic regression analyses were presented as odds ratios (ORs) with 95% confidence intervals (CIs). Also, it was carried out a complete-case analysis. The missing data were as follows: physical activity (*n* = 5312; 23.0%), chronic conditions (*n* = 0; 0%), gender (*n* = 0; 0%), age (*n* = 0; 0%), BMI (*n* = 1070; 4.6%), education level (*n* = 0; 0%), the fact of living as a couple (*n* = 139; 0.6%), smoking (*n* = 22; 0.1%), and alcohol consumption (*n* = 26; 0.1%). *p* < 0.05 was the statistical significance level in this study.

## 3. Results

The sample for this study was composed of 23,089 adults (age range: 15–103 years; average age 53.4 ± 18.9 years; 54.1% females). The number of people with each one of the 32 chronic conditions analysed was: 1. Hypertension [*n* = 6244]; 2. Myocardial infarction [*n* = 530]; 3. Angina, Coronary heart disease [*n* = 503]; 4. Other heart disease [*n* = 1561]; 5. Varicose veins (legs) [*n* = 2862]; 6. Arthrosis [*n* = 5234]; 7. Chronic back pain (cervical) [*n* = 3874]; 8. Chronic back pain (lumbar) [*n* = 5077]; 9. Chronic allergy (allergic asthma excluded) [*n* = 3741]; 10. Asthma (allergic asthma included) [*n* = 1381]; 11. Chronic bronchitis, emphysema, COPD [*n* = 1154]; 12. Diabetes [*n* = 2266]; 13. Stomach/duodenal ulcer [*n* = 1024]; 14. Urinary incontinence [*n* = 1357]; 15. High cholesterol [*n* = 5462]; 16. Cataracts [*n* = 2878]; 17. Chronic skin problems [*n* = 1407]; 18. Chronic constipation [*n* = 1045]; 19. Cirrhosis, liver dysfunction [*n* = 338]; 20. Depression [*n* = 2464]; 21. Chronic anxiety [*n* = 2078]; 22. Other mental problems [*n* = 503]; 23. Stroke [*n* = 486]; 24. Migraine or frequent headache [*n* = 2332]; 25. Haemorrhoids [*n* = 1720]; 26. Malignant tumors [*n* = 1720]; 27. Osteoporosis [*n* = 1180]; 28. Thyroid problems [*n* = 1617]; 29. Kidney problems [*n* = 1131]; 30. Prostate problems (males) [*n* = 1006]; 31. Menopausal problems (females) [*n* = 691]; and 32. Permanent injuries caused by accident [*n* = 1443].

**Figure 1:**
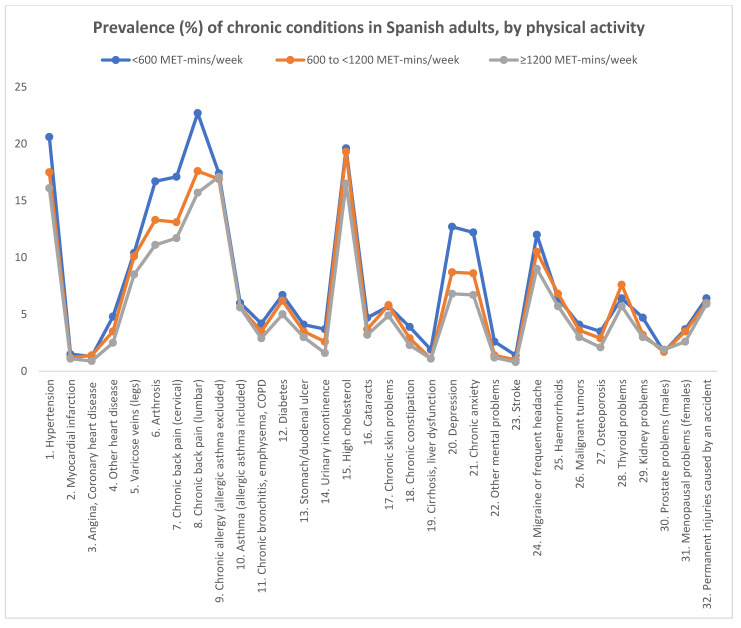
Prevalence of chronic conditions in Spanish adults by physical activity.

The analysis of the prevalence of chronic conditions by level of physical activity showed a clear trend, in such a way that the highest prevalence of chronic conditions was in the group doing less than 600 MET-min/week of physical activity, while the lowest prevalence of chronic conditions was in the group doing at least 1200 MET-min/week of physical activity. The highest prevalence of chronic conditions was found in the group doing less than 600 MET-min/week of physical activity in 28 of the 32 conditions analysed, namely all the conditions except angina, coronary heart disease; chronic skin problems; haemorrhoids; and prostate problems (males). The lowest prevalence was found in the group doing at least 1200 MET-min/week for 30 of the 32 conditions analysed, namely all the conditions except chronic allergy (allergic asthma excluded) and prostate problems (males) (Table 1, Figure 1).

Adjusted multivariable logistic regression analyses showed that less than 600 MET-min/week of physical activity was significantly associated with a higher risk of 19 chronic conditions: hypertension; other heart diseases; arthrosis; chronic back pain (cervical); chronic back pain (lumbar); stomach/duodenal ulcer; urinary incontinence; high cholesterol; cataracts; chronic constipation; cirrhosis, liver dysfunction; depression; chronic anxiety; other mental problems; stroke; migraine or frequent headache; malignant tumours; osteoporosis; and kidney problems. All these significant associations, except for hypertension, were also maintained in those doing less than 1200 MET-min/week (Table 2). 

## 4. Discussion

### 4.1. Interpretation of Findings

Our study results are in line with previous studies that have analysed the association between physical activity and the main types of chronic conditions [4,8,9,11]. However, we have found significant associations not only in the main types of chronic conditions, like in the systematic review of Bullard et al. [25] but also in other chronic diseases, with a total of 19 chronic conditions in the adjusted analyses. Most previous studies analysing the association between physical activity and chronic conditions focused on one type of chronic condition or a small group of these conditions, in contrast to the present study that analysed 32 chronic conditions. However, our results can be compared with those of Vancampfort et al. [26], who conducted a big study analysing a total of nine chronic conditions. Like in the present study, Vancampfort et al. [26] found that those with chronic conditions were significantly less physically active.

Although each chronic condition has particular characteristics, there are plausible pathways that may explain the observed associations between low levels of physical activity and all these conditions. It is possible that the multiple benefits of physical activity act as a barrier against the risk of chronic conditions. As reported by the World Health Organization [4], physical activity has significant health benefits for hearts, bodies, and minds. Similarly, the Centers for Disease Control and Prevention underline that regular physical activity helps improve overall health, fitness, and quality of life [27]. Previous research has shown that regular physical activity might be able to offset disease-causing cellular damage and slow the ageing process; although ageing cannot be completely stopped, staying active can slow down the destructive processes that lead to disease and immobility [28,29]. In fact, physical activity can rejuvenate cells, extending a lifespan because it improves muscle health by renewing the mitochondria [30,31] and attenuating skeletal muscle epigenetic ageing [32,33]. Finally, regular physical activity is associated with maintaining longer telomeres [34], improving biological age, and may increase individuals’ regenerative potential [35].

### 4.2. Implication of Study Findings

The findings of this study indicate that doing physical activity is an adequate way to prevent chronic conditions. In this study, the higher risk of multiple chronic conditions was found not only in those who did less than 600 MET-min/week of physical activity but also in those who did less than 1200 MET-min/week, with the lowest prevalence of chronic conditions in the people who did more than 1200 MET-min/week of physical activity. The implications of these findings are very important because they suggest that, in order to prevent chronic conditions, international physical activity guidelines should recommend that the amount of weekly physical activity in adults should be higher than 1200 MET-min/week of physical activity, equivalent to the upper limit of the current WHO physical activity guidelines [4,5,6], instead of 600 MET-min/week (equivalent to the lowest limit of the current WHO physical activity guidelines).

### 4.3. Strengths and Limitations

This study has important strengths. First, the use of a large representative sample of Spanish adults with good age and gender distribution. Second, the use of a validated, reliable, and internationally recognized questionnaire to assess physical activity. Third, the analysis of a total of 32 chronic conditions. However, this study also presented some limitations, which should be considered when interpreting our results. The first limitation was that the study was self-reported, and it is possible that this fact introduced recall bias in our results. Another limitation refers to the question that was used to evaluate if the participants suffered from chronic conditions, as the question “have you ever” allows for the possibility that respondents had the conditions before the existence of physical inactivity. Third, the study was cross-sectional in nature, and thus the direction of association could not be established. Consequently, we recommend future longitudinal studies that address the previous limitations, especially longitudinal randomized clinical trials. 

## 5. Conclusions

The results of this study clearly show that higher physical activity is a protective factor against the risk of suffering from chronic conditions, with the lowest prevalence of chronic conditions in people doing more than 1200 MET-min/week of physical activity. According to our results, there is a higher risk of multiple chronic conditions not only in those doing less than 600 MET-min/week of physical activity but also in those doing less than 1200 MET-min/week. These results suggest that international recommendations for physical activity should not use the cutoff of 600 MET-min/week as the minimum physical activity recommended, with at least 1200 MET-min/week of physical activity being a cutoff more appropriate to prevent the risk of chronic conditions. 

## Figures and Tables

**Table 1:** Prevalence of chronic conditions in Spanish adults by physical activity.

Chronic Conditions	MET-min/Week of Physical Activity		
	<600	600 to <1200	≥1200
1. Hypertension	1105 (20.6)	519 (17.5)	1520 (16.1)
2. Myocardial infarction	82 (1.5)	35 (1.2)	103 (1.1)
3. Angina, Coronary heart disease	69 (1.3)	41 (1.4)	89 (0.9)
4. Other heart disease	257 (4.8)	104 (3.5)	236 (2.5)
5. Varicose veins (legs)	559 (10.4)	299 (10.1)	800 (8.5)
6. Arthrosis	897 (16.7)	393 (13.3)	1053 (11.1)
7. Chronic back pain (cervical)	919 (17.1)	388 (13.1)	1108 (11.7)
8. Chronic back pain (lumbar)	1219 (22.7)	520 (17.6)	1481 (15.7)
9. Chronic allergy (allergic asthma excluded)	932 (17.4)	500 (16.9)	1617 (17.1)
10. Asthma (allergic asthma included)	322 (6.0)	165 (5.6)	533 (5.6)
11. Chronic bronchitis, emphysema, COPD	223 (4.2)	103 (3.5)	275 (2.9)
12. Diabetes	359 (6.7)	183 (6.2)	472 (5.0)
13. Stomach/duodenal ulcer	220 (4.1)	105 (3.5)	280 (3.0)
14. Urinary incontinence	198 (3.7)	76 (2.6)	151 (1.6)
15. High cholesterol	1051 (19.6)	570 (19.3)	1561 (16.5)
16. Cataracts	253 (4.7)	109 (3.7)	302 (3.2)
17. Chronic skin problems	304 (5.7)	171 (5.8)	465 (4.9)
18. Chronic constipation	210 (3.9)	85 (2.9)	213 (2.3)
19. Cirrhosis, liver dysfunction	101 (1.9)	34 (1.1)	106 (1.1)
20. Depression	680 (12.7)	256 (8.7)	641 (6.8)
21. Chronic anxiety	657 (12.2)	255 (8.6)	631 (6.7)
22. Other mental problems	141 (2.6)	40 (1.4)	109 (1.2)
23. Stroke	74 (1.4)	30 (1.0)	76 (0.8)
24. Migraine or frequent headache	645 (12.0)	310 (10.5)	855 (9.0)
25. Haemorrhoids	344 (6.4)	202 (6.8)	535 (5.7)
26. Malignant tumours	219 (4.1)	108 (3.6)	288 (3.0)
27. Osteoporosis	187 (3.5)	87 (2.9)	197 (2.1)
28. Thyroid problems	345 (6.4)	224 (7.6)	543 (5.7)
29. Kidney problems	252 (4.7)	94 (3.2)	284 (3.0)
30. Prostate problems (males)	93 (1.7)	51 (1.7)	183 (1.9)
31. Menopausal problems (females)	197 (3.7)	105 (3.5)	247 (2.6)
32. Permanent injuries caused by an accident	344 (6.4)	178 (6.0)	557 (5.9)

Results presented as frequencies (valid%).

**Table 2:** Association between low levels of physical activity (exposure) and chronic conditions (outcome) in Spanish adults, estimated by multivariable logistic regression.

Chronic Conditions	<600 MET-min/Week	<1200 MET-min/Week
1. Hypertension	1.130 (1.026–1.244) *	1.080 (0.987–1.182)
2. Myocardial infarction	1.171 (0.782–1.574)	1.209 (0.912–1.601)
3. Angina, Coronary heart disease	1.082 (0.795–1.473)	1.323 (0.987–1.774)
4. Other heart diseases	1.576 (1.324–1.877) ***	1.569 (1.318–1.867) ***
5. Varicose veins (legs)	1.022 (0.910–1.147)	1.026 (0.920–1.144)
6. Arthrosis	1.369 (1.234–1.520) ***	1.205 (1.091–1.332) ***
7. Chronic back pain (cervical)	1.348 (1.225–1.484) ***	1.195 (1.090–1.310) ***
8. Chronic back pain (lumbar)	1.383 (1.270–1.506) ***	1.254 (1.156–1.361) ***
9. Chronic allergy (allergic asthma excluded)	1.059 (0.970–1.157)	1.029 (0.949–1.116)
10. Asthma (allergic asthma included)	1.065 (0.924–1.227)	1.025 (0.899–1.169)
11. Chronic bronchitis, emphysema, COPD	1.182 (0.990–1.413)	1.166 (0.983–1.383)
12. Diabetes	1.001 (0.863–1.160)	1.062 (0.924–1.222)
13. Stomach/duodenal ulcer	1.218 (1.021–1.452) *	1.214 (1.026–1.437) *
14. Urinary incontinence	1.677 (1.367–2.058) ***	1.721 (1.396–2.123) ***
15. High cholesterol	1.102 (1.005–1.208) *	1.117 (1.026–1.217) *
16. Cataracts	1.324 (1.110–1.579) **	1.204 (1.016–1.426) *
17. Chronic skin problems	1.086 (0.939–1.257)	1.129 (0.986–1.293)
18. Chronic constipation	1.497 (1.240–1.808) ***	1.346 (1.116–1.622) **
19. Cirrhosis, liver dysfunction	1.595 (1.222–2.081) ***	1.410 (1.083–1.837) *
20. Depression	1.609 (1.435–1.803) ***	1.475 (1.317–1.651) ***
21. Chronic anxiety	1.573 (1.405–1.760) ***	1.473 (1.318–1.647) ***
22. Other mental problems	1.786 (1.391–2.294) ***	1.679 (1.301–2.167) ***
23. Stroke	1.401 (1.025–1.914) *	1.405 (1.033–1.910) *
24. Migraine or frequent headache	1.206 (1.084–1.343) ***	1.154 (1.042–1.278) **
25. Haemorrhoids	1.016 (0.885–1.166)	1.075 (0.946–1.222)
26. Malignant tumors	1.267 (1.062–1.513) **	1.226 (1.035–1.451) *
27. Osteoporosis	1.481 (1.206–1.819) ***	1.355 (1.110–1.656) **
28. Thyroid problems	0.935 (0.813–1.076)	1.017 (0.894–1.158)
29. Kidney problems	1.375 (1.160–1.630) ***	1.250 (1.059–1.475) **
30. Prostate problems (males)	1.042 (0.800–1.359)	1.083 (0.852–1.378)
31. Menopausal problems (females)	1.100 (0.908–1.331)	1.091 (0.908–1.309)
32. Permanent injuries caused by an accident	1.073 (0.936–1.231)	1.082 (0.952–1.228)

Results presented as odds ratio (95% confidence interval). * *p* < 0.05. ** *p* < 0.01. *** *p* < 0.001. Models adjusted for gender, age, BMI, education level, living as a couple, smoking, and alcohol consumption.

## Data Availability

Data supporting the findings of this study are available from the corresponding author upon reasonable request.
